# The Creatine Transporter Unfolded: A Knotty Premise in the Cerebral Creatine Deficiency Syndrome

**DOI:** 10.3389/fnsyn.2020.588954

**Published:** 2020-10-23

**Authors:** Clemens V. Farr, Ali El-Kasaby, Michael Freissmuth, Sonja Sucic

**Affiliations:** Institute of Pharmacology, Center of Physiology and Pharmacology, Medical University of Vienna, Vienna, Austria

**Keywords:** creatine, intellectual disability, creatine transporter 1, creatine transporter deficiency, protein misfolding, pharmacochaperoning, SLC6A8

## Abstract

Creatine provides cells with high-energy phosphates for the rapid reconstitution of hydrolyzed adenosine triphosphate. The eponymous creatine transporter (CRT1/SLC6A8) belongs to a family of solute carrier 6 (SLC6) proteins. The key role of CRT1 is to translocate creatine across tissue barriers and into target cells, such as neurons and myocytes. Individuals harboring mutations in the coding sequence of the human CRT1 gene develop creatine transporter deficiency (CTD), one of the pivotal underlying causes of cerebral creatine deficiency syndrome. CTD encompasses an array of clinical manifestations, including severe intellectual disability, epilepsy, autism, development delay, and motor dysfunction. CTD is characterized by the absence of cerebral creatine, which implies an indispensable role for CRT1 in supplying the brain cells with creatine. CTD-associated variants dramatically reduce or abolish creatine transport activity by CRT1. Many of these are point mutations that are known to trigger folding defects, leading to the retention of encoded CRT1 proteins in the endoplasmic reticulum and precluding their delivery to the cell surface. Misfolding of several related SLC6 transporters also gives rise to detrimental pathologic conditions in people; e.g., mutations in the dopamine transporter induce infantile parkinsonism/dystonia, while mutations in the GABA transporter 1 cause treatment-resistant epilepsy. In some cases, folding defects are amenable to rescue by small molecules, known as pharmacological and chemical chaperones, which restore the cell surface expression and transport activity of the previously non-functional proteins. Insights from the recent molecular, animal and human case studies of CTD add toward our understanding of this complex disorder and reveal the wide-ranging effects elicited upon CRT1 dysfunction. This grants novel therapeutic prospects for the treatment of patients afflicted with CTD, e.g., modifying the creatine molecule to facilitate CRT1-independent entry into brain cells, or correcting folding-deficient and loss-of-function CTD variants using pharmacochaperones and/or allosteric modulators. The latter justifies a search for additional compounds with a capacity to correct mutation-specific defects.

## The Creatine Transporter Deficiency (CTD) Syndrome

Creatine transporter deficiency (CTD) is one of the known genetic causes of cerebral creatine deficiency syndromes (CCDS). In CTD, creatine is incapable of entering the brain cells *via* the designated creatine transporter 1 (CRT1). The remaining two CCDS disorders are caused by deficiencies in the enzymes arginine: glycine amidinotransferase (AGAT) and guanidinoacetate methyltransferase (GAMT), which are required for creatine synthesis in the body. All three deficiencies result in the absence of creatine in the brain. Because creatine is fundamental to normal brain development and function, the most frequent and core phenotype of CTD, and CCDS overall, is marked intellectual disability. Apart from this predicament, the clinical picture of the disease manifests in a complex spectrum of additional clinical symptoms including epileptic seizures, development delay (language and walking), behavior problems (autism, attention deficit hyperactivity disorder (ADHD), motor dysfunction (lack of coordination and dystonia), failure to thrive, and gastrointestinal problems (neonatal feeding difficulties, vomiting, constipation and ulcers).

AGAT and GAMT deficiencies affect males and females comparably, as they are inherited in an autosomal recessive manner. CTD, on the other hand, is an X-linked disease and as such predominantly impacts the male population. CTD is caused by mutations in the *SLC6A8* gene (mapped to Xq28), encoding the CRT1 protein. The mutations give rise to CRT1 variants, which dramatically reduce or abolish creatine uptake activity. To date, over 80 pathogenic variants have been identified in the human CRT1 gene. Detailed clinical indications have already been reported for specific point mutations (Table 1). The severity of CTD appears to correlate to residual CRT1 activity; e.g., A404P and P544L, both maintained 5–15% of wild type transporter creatine uptake levels in patient fibroblasts (Mancini et al., 2005; Alcaide et al., 2010) and HEK293 cells (El-Kasaby et al., 2019), and manifested in moderate intellectual disability. The fully inactive P382L variant, on the other hand, elicited a severe form of CTD in the afflicted patients (Mercimek-Mahmutoglu et al., 2009). According to van de Kamp and colleagues, the prevalence of CTD in males with intellectual disability lies between 0.4 and 1.4% (van de Kamp et al., 2014). The estimated carrier prevalence of CTD in females in the general population is a minimum of 0.024% (DesRoches et al., 2015). The true prevalence of CTD worldwide is probably underestimated, partly due to misdiagnosis (e.g., some patients diagnosed with autism, ADHD, or unexplained intellectual disability). Intellectual disability was estimated to have a prevalence of 1% in the general population (McKenzie et al., 2016), and although CTD is a rare disease, it is a significant and underappreciated cause of this condition.

**Table 1:** Point mutations in human creatine transporter 1 (CRT-1) with the consequent CTD phenotypes.

Variant	Clinical phenotype	References
P31L	Seizures, speech and motor delay, and thalamus atrophy	Rostami et al. (2020)
G87R	Intellectual disability	Rosenberg et al. (2004)
G132V	Intellectual disability	Lion-François et al. (2006)
R207W	Global developmental delays, hypotonia, intellectual disability, and language apraxia	Ardon et al. (2016)
G253R	Mild intellectual disability and language delay	Battini et al. (2011)
N331K	Speech delay, seizures, and hyperactivity	Mencarelli et al. (2011)
C337W	Intellectual disability	Rosenberg et al. (2004)
G356V	Epilepsy and mild intellectual disability	Mercimek-Mahmutoglu et al. (2009)
G381R	Intellectual disability, seizures, speech, and behavioral disturbance, hypotonia, and gastrointestinal problems	Hahn et al. (2002)
P382L	Severe intellectual disability, speech delay, behavioral problems, and epilepsy	Mercimek-Mahmutoglu et al. (2009)
P390L	Intellectual disability, learning difficulties, seizures, hyperactive and impulsive behavior	Rosenberg et al. (2004) and Heussinger et al. (2017)
R391W	Seizures, hyperactivity, aggressiveness, and hyperphagia	Mencarelli et al. (2011)
T394K	Intellectual disabilities, severe developmental delay and speech impairment, seizures, and mild scale autism	Wang et al. (2018)
A404P	Mild psychomotor retardation and language impairments	Alcaide et al. (2010)
G424D	Speech and language delay, learning difficulties, mild autistic features, social anxiety and attention deficit, aggressiveness, impulsiveness, and hyperactivity	Puusepp et al. (2010)
G466R	Developmental delay, dystonia, no speech, and epilepsy	Alcaide et al. (2011)
D474G	Mild intellectual disability and occasional febrile seizures	Bruun et al. (2018)
C491W	Generalized tonic-clonic seizures	Lion-François et al. (2006)
M510K	Moderate intellectual disability, antiepileptic drug-responsive seizures, hypotonia, and dysarthria	Bruun et al. (2018)
P544L	Moderate intellectual disability, generalized hypotonia, delayed language, and speech skills, and multifocal epileptic waves	Mancini et al. (2005)
P554L	Intellectual disability, hypotonia, intellectual disability, severe speech delay, seizures, autism, and epilepsy	Rosenberg et al. (2004) and Nozaki et al. (2015)
G561A	Intellectual disability	Kato et al. (2014)
F315del	Intellectual disability, epilepsy, autism, and speech delay	Fons et al. (2009)
N336del	Intellectual disability, seizures, and motor dyspraxia	Battini et al. (2007)
I347del	Moderate intellectual disability, aggressive behavior, and seizures	Clark et al. (2006)
F354del	Intellectual disability	Betsalel et al. (2008)
F360del	Intellectual disability, epilepsy, autism, and speech delay	Fons et al. (2009) and Sempere et al. (2009)
F408del	Intellectual disability, epilepsy, autism, seizure, speech and language delay and a reduced interest in the surroundings	Bizzi et al. (2002) and Póo-Argüelles et al. (2006)

*Only those CTD variants with identified clinical symptoms are listed, according to the reports available in the literature to date*.

About 20–30% of all CTD variants are thought to be caused by *de novo* mutations (DesRoches et al., 2015). The actual frequency of *de novo* mutations is unknown, but the emerging state-of-the-art genetic tests, such as trio (father, mother, child) based exome sequencing (Kreiman and Boles, 2020) ought to improve the diagnostic approaches for CTD. In the occurrence of CTD mutations, all males, and about 50% of the affected females, typically display intellectual and cognitive dysfunction (DesRoches et al., 2015). Despite CTD being an X-linked disease, females carrying heterozygous pathogenic mutations have been identified, albeit the extent of disease severity is comparatively mild, e.g., female relatives of CTD patients harboring the G381R and R514X mutations have only minor neuropsychological impairments. It is not entirely clear why some women are protected/asymptomatic carriers, while others are susceptible and develop CTD. The source of variability among female carriers can be rationalized by several factors. (i) Lyonisation or random X-inactivation (Davidson, 1964; Migeon, 2020), may clarify how a phenotypic consequence of the X chromosome can be manifested in mammalian females and males alike, despite females having two somatic X chromosomes and males only one. In females, one X chromosome undergoes inactivation during early embryonic development, such that the end quantities of X-linked gene products in females and males are similar. X-inactivation is random and incomplete, and some segments of the silenced X chromosome can escape inactivation. Females can thus be genetic mosaics, i.e., pathogenicity is ameliorated because the variant is not expressed in all their cells, in contrast to the hemizygous males, who express the pathogenic variant allele in every cell (Migeon, 2020). (ii) In some cases, females manifest the disease because the pathogenic variant interacts with the healthy allele in a dominant-negative manner. (iii) Clinical phenotypes can also be shaped by so-called modifier genes, which alter the expression of other genes, e.g., specific modifiers can differentially affect the incidence, severity as well as the time of onset of target gene diseases, as already reported for e.g., cystic fibrosis, epilepsy and sickle cell disorder (Riordan and Nadeau, 2017). Nonetheless, no such modifier genes have been identified in female carriers of pathogenic CRT1 mutations to date, and future studies may clarify the contribution of this mechanism to the clinical variability among female carriers of CTD.

## Unraveling the Intricate Clinical Phenotype of CTD

### The Brain as a Core Source of the Most Severe Symptoms in CTD

**Figure 1:**
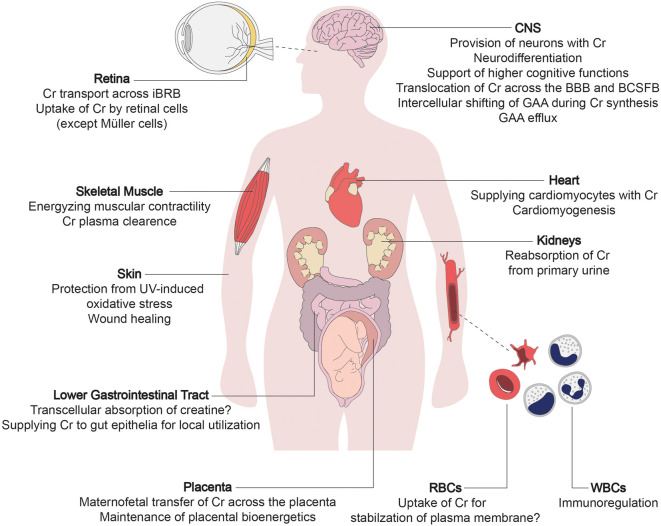
The multi-layered physiological roles of creatine transporter 1 (CRT1). The primary function of creatine transporter 1 (CRT1) is to refill cellular creatine reserves, to compensate for its steady degradation (1.7% daily), in end-consumer cells incapable of self-synthesizing sufficient amounts of creatine (i.e., neurons, myocytes, and cardiomyocytes). CRT1 orchestrates creatine flux throughout the body by tuning the amount of creatine extracted from the blood by individual organs. CRT1 plays a key role in providing the brain with creatine, essential to higher cognitive functions. It shifts creatine across tissue barriers like the kidney tubules, the blood-brain barrier (BBB), and the inner blood-retinal barrier. It thus mediates reabsorption of creatine from the primary urine and facilitates the maintenance of cerebral and retinal tissue energetics, respectively. At the placenta, CRT1 takes up creatine, crucial for embryofetal development. It also supports the functioning and integrity of the intestinal barrier, and is an immunoregulator in leukocytes and stabilizes erythrocyte plasma membranes. Cr, creatine; BBB, blood-brain barrier; BCSFB, blood-cerebrospinal fluid barrier; iBRB, inner blood-retinal barrier; GAA, guanidinoacetate; RBC, red blood cell; WBC, white blood cell.

All individuals afflicted with CTD suffer from mild to severe impairment of brain function, which is explicable considering the lack of creatine supply to brain cells. Apart from the robust expression in the brain, CRT1 is also present in other tissues, justifying some of the peripheral symptoms of CTD in the affected individuals (Figure 1). In the human brain, CRT1 shows the highest expression in the pyramidal neurons in the cerebral cortex, Purkinje cells of the cerebellar cortex, hippocampus, and motor neurons of the somatic motor and visceromotor cranial nerve nuclei and the ventral horn of the spinal cord (Lowe et al., 2015). CTD impinges on many cerebral tasks, apart from the vital functions supported by the brainstem, which are selectively spared. The brainstem is phylogenetically a very old part of the brain, implying that CRT1 expression peaked only recently in hominid phylogeny. Moreover, CRT1 expression is much higher in the human brain compared to other primates, leading to the hypothesis that the energetic demand of encephalization (i.e., the emergence of intellect) was met by increased meat (that is, creatine) consumption during evolution (Pfefferle et al., 2011). Intellectual disability epitomizes the unifying symptom in all CTD cases, while other neural and extraneural functions remain affected to variable degrees.

Many of the brain symptoms of CTD were probed using mouse models of the disease. The hippocampus is a crucial neuroanatomical component in cognitive deficits related to CTD. Hippocampi of CRT1-deficient mice had lipofuscin accumulation, reduced volume, and reduced adult neurogenesis (Baroncelli et al., 2016). CRT1-knockout mice mirrored the cognitive phenotypes of CTD in people. The mice displayed poor performance in Morris Water Maze, novel object recognition, radial arm- and Y-maze tests (Skelton et al., 2011; Kurosawa et al., 2012; Baroncelli et al., 2014, 2016; Udobi et al., 2018; Molinaro et al., 2019). Collectively, these studies ratified the link between dysfunctional CRT1 and learning disabilities and impairments in declarative long-term as well as working memory. Importantly, it may also be concluded that the employed animal models accurately imitate the predominant human CTD symptoms (van de Kamp et al., 2014). Cognitive deficiencies deteriorated with age in a murine model, alluding to the indispensable role of CRT1 in postnatal development of cognitive skills (Baroncelli et al., 2016; Molinaro et al., 2019). Conditional deletion of CRT1 in mice at postnatal day 5 led to cognitive deficits, whereas deletion at day 60 did not (Udobi et al., 2019). Therefore, cognitive symptoms in CTD may be preventable. Pre-symptomatic treatment with creatine in AGAT- and GAMT-deficiencies, in fact, did prevent further development of central nervous system symptoms in CCDS patients (Battini et al., 2006; Schulze et al., 2006).

Epileptic seizures are one of the most severe clinical symptoms of CTD, with many patients suffering from treatment-resistant epilepsies. The seizure phenotype has yet to be mimicked in the genetic mouse models of CTD. Likewise, autism is frequent among CTD patients (van de Kamp et al., 2014), but not entirely reflected in CRT1-deficient mice, possibly owing to the general difficulty of modeling autistic traits in rodents (Skelton et al., 2011; Baroncelli et al., 2016). ADHD is another common symptom of CTD (van de Kamp et al., 2014). CRT1-knockout mice exhibited hyperactive behavior (Kurosawa et al., 2012; Udobi et al., 2018, 2019; Molinaro et al., 2019). Hyperactivity was one of the few symptoms also observed in female mice (Hautman et al., 2014), closely reflecting the clinical portrait of CTD in people. The dopaminergic system was proposed to play a part in CTD-linked hyperactivity: CRT1-deletion from dopaminergic neurons was sufficient to trigger hyperactivity in mice (Abdulla et al., 2020a). However, this is difficult to reconcile with the low expression levels of CRT1 in dopaminergic neurons in people (Lowe et al., 2015). CTD may be linked to incoherent synaptic transmission, evident from patient fibroblasts having a discrepant expression pattern of synapse-linked genes (Nota et al., 2014). Studies of the monoaminergic system in the brains of CRT1 knockout mice revealed no major changes in dopamine, norepinephrine, or serotonin levels (Skelton et al., 2011; Abdulla et al., 2020b). The amount of γ-aminobutyric acid (GABA) and its metabolites was unfortunately not measured, although the GABAergic system is likely to be involved in CTD-related epilepsy. CRT1-deficient mice have less vesicular GABA transporters (Baroncelli et al., 2016). Also, succinate-semialdehyde dehydrogenase and GABA transaminase were upregulated, along with mitochondrial pyridoxal kinase in CRT1-deficient mouse brains (Giusti et al., 2019). Increased pyridoxal kinase activity in CRT1 knockout mice translates into higher levels of pyridoxal phosphate, a potent modulator of GABA synthesis, degradation, and uptake (Norris et al., 1983; Jung et al., 2019). Moreover, one clinical study reported drastically higher levels of guanidino acetic acid (GAA) in CTD patient brain tissues, with virtually no detectable creatine (Sijens et al., 2005). Increased GAA levels were also detected in CRT1-knockout mice (Baroncelli et al., 2016; Udobi et al., 2018). GAA is a biosynthetic precursor of creatine and may impose on the GABAergic signaling in CTD: it activates GABA_A_ receptors (Neu et al., 2002) and decreases the expression of glutamate decarboxylase and GABA_B_ receptors (Hanna-El-Daher et al., 2015). Wild type CRT1 transports GAA during cerebral creatine synthesis (Braissant et al., 2010) and out of the cerebrospinal fluid (Tachikawa et al., 2008). Accordingly, the lack of functional CRT1 in CTD could lead to cerebral accumulation of GAA which, being an endogenous convulsant, may trigger epileptic seizures in CTD patients.

### Skeletal and Heart Muscles in CTD

CTD patients frequently display a slender physique and hypotonia (van de Kamp et al., 2014). In CRT1-deficient mice, muscles are reduced in size and are also entirely devoid of creatine (Russell et al., 2014; Stockebrand et al., 2018). Muscle fiber atrophy, or rather reduced myocyte numbers, explain the poorly developed muscle mass and low endurance experienced by many CTD patients. Creatine content in the skeletal muscles of two CTD patients was surprisingly not declined (De Grauw et al., 2003; Pyne-Geithman et al., 2004), possibly due to residual CRT1 function in these particular variants (e.g., delF107). This was also the case for some murine models of the disease and may originate from compensatory upregulation of AGAT in the skeletal muscle (Russell et al., 2014; Stockebrand et al., 2018). Moreover, CRT1-independent creatine uptake may occur in skeletal muscles due to the absence of a semipermeable endothelial barrier. In contrast, the brain is enveloped by the blood-brain-barrier, which does not permit the entry of hydrophilic creatine molecules, making the cerebral symptoms of CTD far more severe than muscular defects. Also, motor function is less affected in CTD mice harboring a brain-specific knockout, as opposed to a global CRT1 knockout (Kurosawa et al., 2012; Udobi et al., 2018; Molinaro et al., 2019).

Creatine promotes myoblast differentiation, although a direct role for CRT1 in embryofetal muscle development remains to be confirmed (Deldicque et al., 2007). Intact CRT1 activity allows for maternal creatine supply during the fetogenesis of AGAT-deficient mice, which is absent in CTD (Stockebrand et al., 2018). Relative to CTD, muscular pathologies in AGAT-deficient mice are less pronounced (Nabuurs et al., 2013). CRT1 is abundantly expressed in lower motor neurons (Mak et al., 2009; Lowe et al., 2015), and its deficiency in CTD may be responsible for the muscular atrophy symptoms in people. Furthermore, reduced CRT1 levels in the primary motor cortex might cause the central motor symptoms such as spastic paresis (Mak et al., 2009; Lowe et al., 2015). To date, only two CTD patients are known to suffer from spasticity (Ardon et al., 2016; Rostami et al., 2020). The motor system is also affected by CRT1-expressing cerebellar Purkinje cells, which rank among the highest CRT1-expressing brain regions (Mak et al., 2009; Lowe et al., 2015). CRT1-deficient mice lack cerebral creatine (Molinaro et al., 2019), and consequently display poor motor learning and coordination (Stockebrand et al., 2018). An outcome of compromised cerebellar function is its contribution to the delayed motor skill development, which occurs in most CTD patients (van de Kamp et al., 2014).

The effects on cardiac muscle have been identified in only a few CTD patients (van de Kamp et al., 2013). Under normal physiological conditions, the heart contains large amounts of creatine and expresses abundant amounts of CRT1. Yet, the heart seems to be one of the least affected organs in CTD (along with the retina, see below). Some CRT1-knockout mice have dramatically reduced heart tissue creatine levels, but do not display overt cardiac pathologies (Skelton et al., 2011; Baroncelli et al., 2014; Stockebrand et al., 2018). The discrepancies in human (and murine) cases of CTD may be down to the following: (i) underdiagnosis, i.e., CTD patients and animal models not undergoing appropriate medical examinations and experimental investigation, respectively; (ii) a later time of onset of the cardiac symptoms; (iii) heart tissues acquiring creatine in a CRT1-independent manner (as described above for skeletal muscles); and (iv) the cardiac phenotype may be contingent on the genetic background, which is consistent with the modifier gene concept. Such protective compensatory mechanisms on the heart add up because CRT1 is vital to cardiac function; i.e., the creatine-phosphocreatine circuit sustains cardiomyocytic bioenergetics (Guzun et al., 2011), and CRT1 plays a role in cardiomyogenesis, evident from enhanced myocardial expression of CRT1 upon cardiac maturation in rats (Fischer et al., 2010).

### Effects on the Sensory Organs

High amounts of CRT1 are present in the retina (Acosta et al., 2005; Country, 2017), cochlea, and the auditory brainstem nuclei (Hiel et al., 1996; Wong et al., 2012). Still, this robust expression pattern does not inevitably provide a cue, as there is no evidence for impaired sight or hearing in CTD patients or in CRT1-deficient mouse models (Skelton et al., 2011). In CTD patients, the impaired hearing was proposed to contribute to the delay in speech development (van de Kamp et al., 2014). However, upon direct evaluation by brainstem auditory evoked potentials, the central auditory pathways appeared intact (Schiaffino et al., 2005; Rostami et al., 2020). Otoacoustic emission indicated hearing loss due to outer hair cell malfunction in one CTD patient (Hathaway et al., 2010). No major visual defects were reported in CTD patients, except for sporadic strabismus (Schiaffino et al., 2005; Yu et al., 2013), which can probably be ascribed to ocular myopathy. Visually evoked potentials were assessed in a single CTD patient with no evidence of pathological changes, while compromised ocular fundus was reported in one out of three cases examined (Schiaffino et al., 2005; Anselm et al., 2006). The lack of the anticipated defects in the retina of CTD cases may ensue from compensatory processes such as cell-autonomous creatine synthesis in retinal neurons and/or glia, which presumably sustain adequate amounts of creatine.

### Further Aspects of CTD: Immunity and Cellular Metabolic Networks

CRT1 supports the balance between two types of macrophage activation (Ji et al., 2019). Furthermore, creatine uptake was recently found to modulate CD8 T-cell response to antigen exposure (Di Biase et al., 2019). CRT1 is expressed in leukocytes (Taii et al., 2020) and macrophages (Loike et al., 1986; Lowe et al., 2015; Heinbockel et al., 2018; Ji et al., 2019). Since CRT1-activity was detected in lymphoblastoid cells derived from a CTD patient, it is conceivable that CRT1 has an immunoregulatory role in the body (Leuzzi et al., 2008). CTD mouse models also exhibit a marked increase in activated microglia (Baroncelli et al., 2016). Moreover, the brains of CRT1-deficient animals show an upregulation in cyclophilin A (Giusti et al., 2019), an immune effector molecule whose secretion is enhanced in many inflammatory conditions (Nigro et al., 2013). Both findings constitute unspecific reactions to cellular damage in CTD. Early and late apoptosis was observed in skin fibroblasts cultured from CTD patients with marked increases in reactive oxygen species levels (Alcaide et al., 2011). This is consistent with creatine having antiapoptotic and antioxidant properties (Lawler et al., 2002; Rahimi et al., 2015). In fact, creatine has been in clinical use for its neuroprotective effects (Sullivan et al., 2000; Sakellaris et al., 2006). Also of interest is a report of a novel CRT1 inhibitor, shown to suppress cancer growth and metastatic progression (Kurth et al., 2018), which implicates CRT1 in cancer and prompts a closer survey of cancer incidence among CTD patients.

Creatine is immersed in the metabolic network of cells. This balance is vulnerable and cells have developed compensatory mechanisms to cope with reduced creatine concentrations, e.g., upregulation of AGAT in kidneys and, albeit to an insufficient extent, in muscles of CRT1-deficient rodents (Russell et al., 2014; Stockebrand et al., 2018). Also, decreases in cellular energy reserves cause a multitude of mitochondrial adaptions in models of CTD. The upregulation of oxidative enzymes was confirmed by proteomic analysis of brain mitochondria in CRT1-knockout mice (Giusti et al., 2019). The increase in mitochondrial density may compensate for the lack of spatial ATP buffering, by decreasing the distance of diffusion (Oudman et al., 2013). Furthermore, inhibition of CRT1 leads to the upregulation of mitochondrial creatine kinase (CK) in striated muscles (Oudman et al., 2013). A rise in mitochondrial respiration in the hippocampus and skeletal muscle fibers was also observed in CRT1-deficient mice, accompanied by increased O_2_ and CO_2_ consumption assessed by whole-body calorimetry (Perna et al., 2016). Lastly, CRT1-deficient cells down-regulate the mitochondrial uncoupling protein 3, which is likely to limit energy dissipation in the form of heat (Stockebrand et al., 2018). Moreover, the AMP-activated protein kinase (AMPK) is activated by low energy levels and drives ATP production *via* multiple pathways. AMPK is activated in CRT1-deficient and β-guanidino propionic acid (GPA, CRT1 inhibitor)-fed animals (Oudman et al., 2013; Stockebrand et al., 2018). This response to low ATP levels likely aggravates the decreased creatine reabsorption from urine, since AMPK negatively regulates CRT1 expression in kidney epithelia (Li et al., 2010). Conversely, AMPK activation increases creatine uptake in cardiomyocytes (Darrabie et al., 2011). AMPK signaling may underlie the upregulation of fatty acid transporter proteins and glucose uptake *via* GLUT4 observed in animal models of CTD (Oudman et al., 2013; Stockebrand et al., 2018). In addition to stimulating the AMPK pathway, reduced creatine uptake triggers the activation of the mitogen-activated protein kinase 1 (MAPK/ERK) pathway (Giusti et al., 2019), and disturbed ERK1/2 activity has already been linked to intellectual disability (Kalkman, 2012; Pucilowska et al., 2015, 2018; Borrie et al., 2017). Many signaling pathways become activated in nerve and other cells, as they cannot meet their energy demands during sustained activity. Thus, changes in AMPK, MAPK/ERK, and other pathways reflect the malfunctioning of the brain and the altered synaptic transmission in CTD. Exploring cellular compensatory mechanisms as opportunities for alternative CTD treatments, though arduous, may be worthwhile.

## Molecular Features of CTD

### CTD Effects on Neuron Cells: CRT1 Out-of-Place

**Figure 2:**
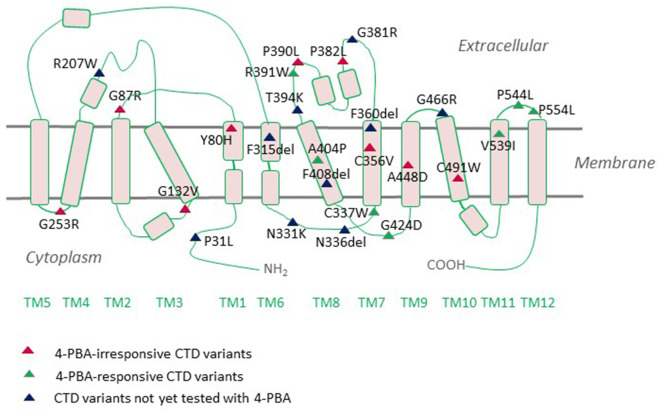
Creatine transporter deficiency (CTD)-associated variants mapped onto a CRT1 topology. CRT1 adopts the common structural fold of SLC6 proteins with 12 transmembrane domains (TMs) and cytoplasmic N- and C-termini. Most of the reported sequence alterations in CTD cluster in the region encompassing TMDs 7 and 8. Only those variants with the ascertained clinical phenotypes are displayed on the topology. Sixteen mutations are known to be folding-deficient. Individual mutants vary in their response to the chemical chaperone 4-PBA.

The neurologic phenotype of CTD is by far the direst of all clinical manifestations of the disease. It is presumed that brain cells depend on local creatine synthesis since the expression levels of CRT1 at the blood-brain barrier (BBB) are comparatively low and the permeability of creatine through the BBB is limited. Indeed, AGAT and GAMT are expressed in neurons, oligodendrocytes, and astrocytes (Braissant et al., 2001, 2010; Tachikawa et al., 2004; Braissant and Henry, 2008). The two enzymes are rarely co-expressed, necessitating the intercellular translocation of the creatine precursor GAA (Braissant et al., 2010). This is most likely achieved by CRT1, which is predominantly expressed in the soma and proximal dendrites of neurons and to a lesser degree in oligodendrocytes and microglia (Braissant et al., 2010; Lowe et al., 2015). Cells with high energetic demands, such as hippocampal pyramidal neurons and Purkinje cells, all express AGAT, GAMT, and CRT1. CRT1 translocates GAA with an affinity 10-fold lower than that of creatine (Tachikawa et al., 2008). Plasma GAA levels are well below creatine levels (Almeida et al., 2004), so extracerebral GAA is not likely to contribute to neuronal creatine synthesis. Hence, in the absence of functional CRTs, brain cells are deprived of their essential energy resource, creatine. However, from a different perspective, one may argue that the capacity of BBB to translocate creatine must not be very high and is capable of sufficiently replacing the lost creatine. Moreover, overexpression of CRT1 in the heart disrupts cardiac bioenergetics, implying that tight regulation of CRT1 expression prevents large increases in creatine concentrations (Wallis et al., 2005). Thus, although the hypothesis that the rodent brain can synthesize creatine is reasonably valid, further experimental evidence is required to grasp these mechanisms. One of the most highly CRT1-expressing cells in the brain are hippocampal neurons, whereby the transporter is delivered to both dendritic and axonal compartments (Dodd et al., 2010). We recently tracked fluorescently-tagged human CRT1 transiently transfected in primary rat hippocampal neurons: the transporter was targetted to the arborizations of neurite extensions and their distal tips. Quite an altered expression pattern was shown by the CTD variant P544L (Table 1), which was confined to the neuronal soma and reached only the proximal neurite segments (El-Kasaby et al., 2019). Thus, defective trafficking of variant CRT1 proteins poses one feasible trigger, at least for some CTD-linked variants. Disease mutations can elicit dysfunction either by interfering with cellular processing (e.g., P544L) or by disrupting protein function. A large number of CTD variants are known to cluster in the region encompassing transmembrane domains (TMDs) 7 and 8, a mutation hot spot (Freissmuth et al., 2018), which delineates the creatine translocation pathway in CRT1 (Figure 2). It is hence not surprising that mutations in this very region abolish transporter activity.

### CTD and Key Insights From SLC6 Transporters Folding and Trafficking

Many CTD variants have been associated with aberrant glycosylation, intracellular retention, and reduced surface expression (Uemura et al., 2017; El-Kasaby et al., 2019). Evidence for proteostatic deficiencies in several CTD mutants was also substantiated by data from the Schlabach group (Salazar et al., 2020). Nascent membrane proteins in the endoplasmic reticulum (ER) are core-glycosylated. After export from the ER, they acquire mature N-linked glycans in the Golgi apparatus. We recently took advantage of glycosidases with different glycan cleavage patterns to demonstrate that 16 CTD-linked variants exhibit an immature glycosylation pattern (El-Kasaby et al., 2019). The mutant proteins also co-localized with the ER-resident chaperone calnexin (El-Kasaby et al., 2019), suggestive of misfolding and ER-retention. Much of the understanding of SLC6 protein folding and trafficking is shaped by paradigmatic studies of the serotonin transporter (SERT) in our laboratory (El-Kasaby et al., 2010; Koban et al., 2015; Kasture et al., 2019). Nascent SLC6 proteins are engaged by various ER-resident chaperones, such as calnexin and a relay of cytosolic chaperones that interact with the transporters’ C-tail regions (Sucic et al., 2016), which serve as molecular folding sensors along the stringent ER quality control cascade. Hence, manipulating different components of the folding trajectory (detailed below) proved effective in the rescue of many misfolded SLC6 transporters, i.e., proper folding permits ER export and trafficking of the client proteins to their designated sites of action in cells.

**Figure 3:**
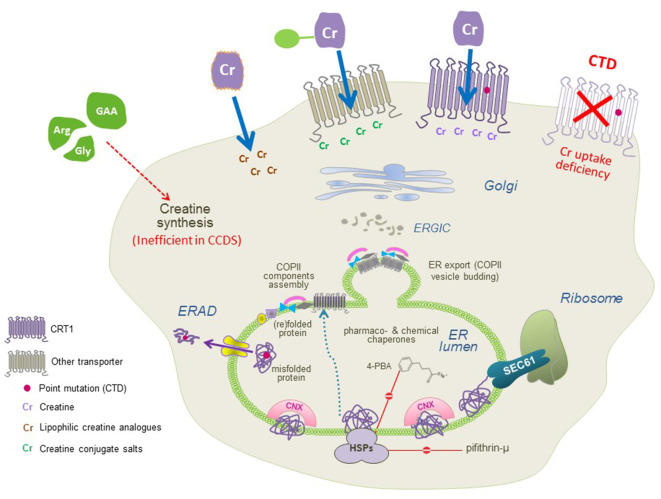
A molecular view of CTD and the putative therapeutic approaches. CTD mutations trigger folding deficits or impair CRT1 substrate uptake activity (both resulting in the lack of functional CRT1 proteins at the cell surface; top right). Three approaches can be used to restore creatine supply in the cells: (1) conjugating creatine with salts, taken up by other transporters; and (2) creating lipophilic creatine analogs, to establish transporter-independent delivery of cyclocreatine, creatine benzyl, and fatty acid esters (top left), or (3) pharmacochaperoning to correct folding defects in CRT1 variants (recovering surface expression and creatine uptake activity of CTD mutant protein, top middle) by treatment with small molecules, that act either directly on the CRT1 itself (i.e., transporter ligands) or manipulate the cellular folding machinery (e.g., heat shock protein inhibitors and chemical chaperones).

The sequence of folding events taking place at the level of ER is meticulous. The ribosome is first recruited to the ER membrane *via* the signal recognition particle (SRP) and the SRP receptor, where the translation arrest is lifted and the transmembrane helices are co-translationally inserted into the translocon/SEC61 channel. In the translocon channel, hydrophobic residues of the nascent chain are shielded from the hydrophilic environment. After the lateral exit of transmembrane segments into the lipid bilayer and N-linked glycosylation, transporter proteins undergo the calnexin cycle on the lumenal side of the ER. On the cytosolic side, the C-terminus is engaged by a heat shock protein relay (e.g., HSP40, 70, and 90) to assist folding and impede premature engagement of the coat protein complex II (COPII)-coat. ER-resident, lumenal chaperones are recruited to the folding intermediates: calnexin recognizes the (re)glycosylated folding intermediates *via* its lectin domain. Upon reaching the minimum energy state (i.e., the stably folded conformation), the chaperones are released. The transporters subsequently form oligomeric complexes and recruit the cognate SEC23/SEC24-dimers (Sucic et al., 2011, 2013) to specific ER exit motifs located on their C-termini. The bow-tie shape of the COPII-component SEC23/SEC24 prompts membrane curvature of the budding COPII vesicles *en route* to the Golgi (Figure 3). Severely misfolded proteins remain bound to calnexin and HSPs and are at last destined for ER-associated degradation (ERAD).

The inheritance pattern of many folding diseases in SLC6 transporters can be rationalized by oligomerization, which takes place at the final stage of folding immediately before the concentrative export of COPII vesicles from the ER compartment. Synthetic ER export-deficient mutations in the C-terminal region of GAT1 elicit a dominant-negative effect on the wild type transporter (Just et al., 2004; Farhan et al., 2007), which also pertains to disease variants: i.e., dominant-negative phenotypes evident for misfolded variants NET-A457P (Hahn et al., 2002) and GLYT2-S510R (Rees et al., 2006; Giménez et al., 2012; Arribas-González et al., 2015). In recessive transmission, misfolded proteins remain trapped by lumenal chaperones in the ER, precluding oligomerization with wild type alleles; e.g., many hyperekplexia variants in GLYT2 (Eulenburg et al., 2006; Rees et al., 2006) or parkinsonism variants in DAT (Kurian et al., 2009, 2011; Ng et al., 2014).

The majority of CTD mutations are located in the hydrophobic core of CRT1 (Figure 2). This is predictable since the interaction of TMDs and the lipid bilayer represents a critical factor in SLC6 transporter folding. In the lipid bilayer, transmembrane segments of nascent SLC6 transporters rearrange to adopt an annular topology, which requires lipid displacement from surfaces of 12 α-helical TMDs, facing one another or away from the translocation pathway (Sucic et al., 2016). The same problem was observed for folding-disease variants in other SLC6 transporters (Freissmuth et al., 2018): e.g., infantile parkinsonism/dystonia in the dopamine transporter (DAT; Kurian et al., 2009, 2011; Ng et al., 2014), epilepsy in the GABA transporter 1 (GAT1), orthostatic intolerance in the transporter for norepinephrine (NET; Hahn et al., 2002) and hyperekplexia in the glycine transporter 2 (GLYT2; Eulenburg et al., 2006; Rees et al., 2006; Arribas-González et al., 2015). Remarkably, certain mutations occur at equivalent residues and give rise to diseases conveyed by the affected transporter: e.g., the folding-deficient missense variant P554L reported both in CRT1 and DAT, triggers severe CTD and Parkinsonism, respectively. Then again, not all of the folding rules, founded on SERT and DAT, necessarily reconcile with CRT1. For example, most SLC6 relatives of CRT1 (DAT, SERT, NET, GAT1, and GLYTs), rely exclusively on their C-terminal domains for folding and trafficking (Farhan et al., 2007; El-Kasaby et al., 2010, 2014; Koban et al., 2015), with their N-termini being virtually dispensable (Sucic et al., 2010; Kern et al., 2017). CRT1 may be an exception to this rule, since the CTD variant P31L, located in the N-terminal region of the transporter, triggers severe functional deficits (Rostami et al., 2020).

## Therapeutic Strategies for Handling CTD

### The Explored Treatment Avenues: Modifying the Creatine Molecule

None of the treatment strategies explored to date have been effective in the long-term management of CTD. Oral supplementation with creatine or creatine precursors (i.e., arginine and glycine) improved the symptoms in some patients with mild CTD symptoms. This therapy appeared beneficial only in patients up to 9 years of age (Dunbar et al., 2014), possibly due to the remnant CRT1 activity in children (Taii et al., 2020) and some CTD variants exhibiting residual creatine uptake (Betsalel et al., 2012; El-Kasaby et al., 2019). Since creatine crosses the BBB very poorly (Ohtsuki et al., 2002; Perasso et al., 2003), some therapeutic approaches rely on boosting intracellular creatine pools using creatine analogs, capable of crossing the BBB and the neuronal plasma membrane (Adriano et al., 2017). Hence, salt-conjugated creatine formulations, which can cross BBB *via* transporters other than CRT1 [e.g., transporters for glucose (GLUTs) or ascorbic acid (SVCT2)], might prove beneficial in enhancing creatine concentrations in the brain. Creatine gluconate and creatine ascorbate increased the creatine content and delayed the population spike disappearance upon anoxia in mouse hippocampal slices, following the inhibition of CRT1 by GPA. But, both compounds failed to improve tissue levels of the energy reserve source, phosphocreatine (Adriano et al., 2017). An additional approach to facilitating creatine translocation into cells is by modifying its lipophilicity; e.g., a more lipophilic cyclocreatine may have therapeutic value, since it is also utilized by mitochondrial CK (Boehm et al., 2003). In CTD mouse models, a 9 week-treatment with a cGMP grade cyclocreatine led to its detection in the brain, hair and claws of the animals. Phosphocyclocreatine was also detected in the brain tissue of the treated mice, along with their spatial learning and memory also normalizing upon cyclocreatine treatment (Kurosawa et al., 2012). In studies carried out in human fibroblast cultures, the uptake of cyclocreatine, and the exchange rate of phosphocyclocreatine, was independent of CRT1, both in wild type and CTD patient cells (Gorshkov et al., 2019). On the other hand, high concentrations of cyclocreatine were associated with reduced ATP levels in both wild type and CTD patient fibroblasts, possibly owing to cyclocreatine dephosphorylation being slower than its phosphorylation (LoPresti and Cohn, 1989). Moreover, the clinical applicability of cyclocreatine is limited, since oral administration in rats set off seizures and showed histopathological changes in the thyroid, testes, and the brain (Kale et al., 2020). Creatine fatty esters were also tested for their ability to elevate cellular creatine levels. Incubating mouse hippocampal slices with creatine benzyl esters or with the phosphocreatine-magnesium acetate complex (at a ratio of 1:1), augmented the tissue creatine content in a CRT1-independent manner, albeit devoid of accompanying boosts in phosphocreatine levels (Lunardi et al., 2006). In CTD patient fibroblasts, dodecyl creatine ester accumulated in amounts comparable to healthy controls, and creatine content rose 20-fold relative to endogenous creatine amounts after a 1-hour treatment in CTD fibroblasts (Trotier-Faurion et al., 2013). Furthermore, lipid nanocapsules were used to overcome the action of plasma esterases, i.e., dodecyl creatine ester incorporated into lipid nanocapsules passed the BBB and increased creatine content in CTD fibroblasts (Trotier-Faurion et al., 2015). This approach may be beneficial in the therapeutic setting. Intracerebroventricular and intranasal administration of dodecyl creatine ester microemulsion to CTD mice, not only increased the creatine content in the brain but was also accompanied by enhanced learning and memory skills, evident from novel object recognition tests (Ullio-Gamboa et al., 2019). Significant efforts are ongoing in clinical programs conducted by Ultragenyx Pharmaceuticals, focusing on the development of new drug treatments for CTD.

### Pharmacochaperoning: Rescue by Folding Correctors

Pharmacochaperoning is another putative treatment strategy for handling CTD, pertinent to the folding-deficient CRT1 variants. Pharmacological and chemical chaperones are small molecules that facilitate the folding and escape of misfolded proteins from the ER, consequently allowing their translocation to the appropriate cellular locations (Welch and Brown, 1996). Pharmacological chaperones are specific ligands (i.e., substrates or inhibitors) which bind to and stabilize the target proteins; e.g., noribogaine efficiently rescued several folding-deficient versions of SERT (El-Kasaby et al., 2014; Koban et al., 2015; Bhat et al., 2017) and parkinsonism variants of DAT (Kasture et al., 2019; Asjad et al., 2017). This is plausible since SLC6 protein folding proceeds *via* the inward-facing transporter conformation and noribogaine binds to the inward-facing conformation of monoamine transporters (Jacobs et al., 2007). Noribogane treatment lowers the energy barrier between folding intermediates and facilitates the progression of transporter proteins along the folding trajectory. Inopportunely, the pharmacology of CRT1 is very modest compared to the rich ligand repertoire of the monoamine transporters (Kasture et al., 2017). Therefore, searching for potential pharmacochaperone (lead) compounds poses a challenge. The identification of lead molecules provides a platform for developing even more efficient pharmacochaperone analogs, which can bind to different protein conformations and hence correct additional misfolded mutants. Apart from the specific ligand, the rescue of folding-deficient proteins can also be achieved by manipulating/relaxing the ER quality control machinery, or by treatment with chemical chaperones (Figure 3). Pifithrin-μ and 17-dimethylaminoethylamino-17-demethoxygeldanamycin (17-DMAG) block HSP70 and HSP90β, respectively, which in turn permits the release of stalled transporter protein complexes (El-Kasaby et al., 2014). HSP blockers *per se* are not ideal chaperone compounds, because they do not act specifically on the misfolded transporters, and are predisposed to unwanted off target effects in a clinical setting.

Pharmacochaperoning has been proposed to work by one of the following four mechanisms: (i) binding to and stabilization of the native state; or (ii) of folding intermediate(s); (iii) suppression of aggregate formation; and (iv) dissolution of aggregates (Marinko et al., 2019). Most compounds, including noribogaine and bupropion (Beerepoot et al., 2016; Asjad et al., 2017), are thought to work *via* the first and/or second mechanism. Tafamidis, used in the treatment of transthyretin amyloidosis, is the only rationally designed drug known to work *via* mechanism (iv). The most effective molecule tested on folding-deficient CRT1 variants was the chemical chaperone 4-phenyl butyric acid (4-PBA) which, at least in part, falls into category (iii). Over a third of the 16 known misfolded CTD variants are amenable to the functional rescue by 4-PBA treatment (Figures 2, 3; El-Kasaby et al., 2019). The rescue effect was also visible in hippocampal neurons expressing the P544L CTD variant, where exposure to 4-PBA promoted its delivery to neurite extensions (El-Kasaby et al., 2019).

Beyond CRT1, the chaperone activity of 4-PBA was also demonstrated on several synthetic misfolded versions of SERT (Fujiwara et al., 2013) and the cystic fibrosis transmembrane conductance regulator (CFTR; Rubenstein and Zeitlin, 2000). In CFTR, 4-PBA exerts its action by stabilizing the misfolded ΔF508-CFTR variant in the ER, subsequently promoting its delivery to the cell surface. 4-PBA also increased the ER-resident core- and mature-glycosylated species of wild type and responsive mutants of CRT1 (El-Kasaby et al., 2019). Interestingly, 4-PBA also helped overcome the dominant-negative effect of a hyperekplexia GLYT2 variant S512R on the wild type transporter, by disturbing oligomer formation between the two versions of the GLYT2 protein (Arribas-González et al., 2015). Classified as a hydrophobic chaperone, 4-PBA interacts with hydrophobic domains of unfolded proteins, thus preventing aggregation [i.e., mechanism (iii) above]. But, this small molecule is incredibly versatile and appears to have many putative mechanisms of action. For instance, it is known to reduce ER stress by downregulating or modulating HSC70 expression (Rubenstein and Zeitlin, 2000), modifying HSP70 levels or blocking histone deacetylases (Cousens et al., 1979), leading to transcriptional regulation of genes in the unfolded protein response system. Interestingly, 4-PBA was approved for clinical use almost 25 years ago. It is used in the chronic management of urea cycle disorders, where it acts as a nitrogen-scavenger assisting kidneys to excrete excess ammonia, as well as in handling sickle cell disease since it activates β-globin transcription (Dover et al., 1994). Over the last decade, the effects of 4-PBA have been tested in many disease models, e.g., Alzheimer’s (Ricobaraza et al., 2009, 2012) and Parkinsons’s disease (Inden et al., 2007; Huang et al., 2017). In light of our recent *in vitro* data on CRT1 variants, translational testing of 4-PBA effects in animal models of CTD would be invaluable, particularly in knock-in animal models harboring folding-defective CRT1 mutations. Since CTD induces irreversible damage already in the early stages of development, it is vital to begin the treatment as early as possible, preferentially throughout the pregnancy.

At the current stage, it is impossible to predict which variants are amenable to rescue by pharmacochaperoning. However, the evolving state-of-the-art computer simulation models, which explore the structural effects of mutations at the atomic level, may convey new insights and serve as a guide for biochemical and pharmacological studies. Such models may hold the predictive power to categorize CTD mutations into (i) folding-deficient variants, which preclude the delivery of CRT1 proteins to the cell surface; and (ii) inactive variants, which are capable of trafficking to the cell surface, but elicit their functional defects as a consequence of conformational alterations in CRT1 (e.g., disruption of the creatine binding site/translocation pathway). In addition to the pharmacochaperone approach, applicable to the misfolded CTD variants, other treatment strategies, which are particularly apt for the second group of mutations, include gene therapy and the use of allosteric modulators. A prominent example of the latter is ivacaftor, which acts as a potentiator of the cystic fibrosis transmembrane conductance regulator (CFTR), by increasing CFTR channel open probability in mutants associated with cystic fibrosis (Van Goor et al., 2009). Allosteric modulators acting on monoamine neurotransmitter transporters also hold potential, in the treatment of various neuropsychiatric disorders (Li et al., 2017; Niello et al., 2020). It is conceivable that effective potentiator molecules can be designed to restore creatine transport in the loss-of-function CRT1 variants linked to CTD.

## Conclusion

Establishing effective long-term treatment schemes for CTD patients poses a major challenge to clinicians and researchers in the field. Current insights into the role(s) of CRT1 in health and disease and the molecular mechanisms underlying CTD have uncovered additional therapeutic avenues that may be worthwhile exploring. Pharmacochaperoning, for instance, ought to be a promising new approach in the treatment of misfolded CRT1 variants associated with CTD. This concept was previously utilized to functionally rescue folding-deficient variants of the human dopamine transporter, which trigger infantile parkinsonism/dystonia, both *in vitro* (heterologous cells) and *in vivo* (*Drosophila melanogaster*). Ongoing endeavors in the rational design and development of novel small molecules with improved capacity for correcting folding deficiencies are of particular interest to those pathologic transporter variants shown to be irresponsive to the existing drugs.

## Author Contributions

SS and CF wrote the manuscript. MF contributed to the conceptual advice. SS, CF, and AE-K created the table and figures. All authors contributed to the article and approved the submitted version.

## Conflict of Interest

The authors declare that the research was conducted in the absence of any commercial or financial relationships that could be construed as a potential conflict of interest.

## Abbreviations

ADHD: attention deficit hyperactivity disorder
AGAT: L-Arginine:glycine amidinotransferase
AMPK: AMP-activated protein kinase
BBB: blood-brain barrier
CCDS: cerebral creatine deficiency syndrome
CFTR: cystic fibrosis transmembrane conductance regulator
CK: creatine kinase
CNX: calnexin
CRT1: creatine transporter-1
CTD: creatine transporter deficiency
DAT: dopamine transporter
ER: endoplasmic reticulum
ERAD: ER-associated degradation
GAA: guanidino acetic acid
GABA: γ-aminobutyric acid
GAMT: guanidinoacetate N-methyltransferase
GAT-1: GABA transporter 1
GLYT: glycine transporter
GPA: β-guanidino propionic acid
NET: norepinephrine transporter
4-PBA: 4-phenyl butyrate
SERT: serotonin transporter
SLC6: solute carrier 6
TMD: transmembrane domain.
